# Establishment and biological characterization of a dermal mesenchymal stem cells line from bovine

**DOI:** 10.1042/BSR20130095

**Published:** 2014-03-31

**Authors:** Tingting Sun, Chao Yu, Yuhua Gao, Chenqiong Zhao, Jinlian Hua, Lianshun Cai, Weijun Guan, Yuehui Ma

**Affiliations:** *Institute of Animal Sciences, Chinese Academy of Agricultural Sciences, Beijing 100193, People's Republic of China; †Institute of Basic Medicine, Jiamusi University, Jiamusi 154007, People's Republic of China; ‡Harbin Institute Of Physical Education, Harbin 150000, People's Republic of China; §College of Veterinary Medicine, Shaanxi Centre of Stem Cells Engineering & Technology, Key Laboratory for Animal Biotechnology of Agriculture Ministry of China, Northwest A & F University, Yangling, Shanxi 712100, People's Republic of China

**Keywords:** bovine, cell culture, dermal mesenchymal stem cell (DMSC), multi-lineage differentiation, BMSC, bone marrow mesenchymal stem cell, DMEM, Dulbecco’s modified Eagle’s medium, DMSC, dermal mesenchymal stem cell, FBS, fetal bovine serum, GAPDH, glyceraldehyde-3-phosphate dehydrogenase, MAP-2, microtubule-associated protein 2, PDT, population doubling time, PPAR-γ, peroxisome-proliferator-activated receptor γ, RT–PCR, reverse transcription–PCR

## Abstract

The DMSCs (dermal mesenchymal stem cells) are multipotent stem cells, which can differentiate *in vitro* into many cell types. Much work has been done on DMSCs from humans, mice, rabbits and other mammals, but the related literature has not been published about these cells in cattle. In this study, we isolated and established the DMSC lines from cattle, thereby initiating further research on these cells, such as growth kinetics, detection of special surface antigen and RT–PCR (reverse transcription–PCR) assays to identify the biological characterization of the cell line. Furthermore, the DMSCs are induced to differentiate into adipocytes, osteoblasts and neural cells *in vitro*. Our results suggest that DMSCs isolated from cattle possess similar biological characteristics with those from other species. Their multi-lineage differentiation capabilities herald a probable application model in tissue engineering and induced pluripotent stem cells.

## INTRODUCTION

The skin is the largest organ of the body and contains a large number of DMSCs (dermal mesenchymal stem cells); they are located in the superficial skin and can be easily obtained; there is a high proliferation rate *in vitro* and a low immunogenicity in cell transplanting [1]. DMSCs were isolated from the dermal tissue in 2000 [2]. DMSCs can differentiated into various cell types *in vitro* such as osteoblasts [3], cartilage cells [4], adipocytes [5], smooth muscle cells [6] and epidermal melanocytes [7].

The characteristics of DMSCs is similar to BMSC (bone marrow mesenchymal stem cell) in self-renewal ability and multi-differentiation. Although DMSCs have not been used as widely as BMSCs in tissue engineering, adult stem cells from the dermal layer of skin are applied to cartilage tissue engineering and may also be a useful cell source for other mesenchymal tissues [4].

Recently the derivation of engineered stem cells or human iPSCs (induced pluripotent stem cells [8]) through the reprogramming of adult fibroblasts is a major advancement in the field of cell therapeutics [9] and regenerative medicine [10]. DMSCs are also considered as better cells in the formation of induced pluripotent stem cells [11]. It has been reported that the human hair follicle's dermal papilla cells are reprogrammed into induced pluripotent stem cells [12].

## MATERIALS AND METHODS

### Experimental animal

A 3–4-month-old Simmental bovine fetus was provided by the Animal Experimental Base Institute of Animal Sciences, Chinese Academy of Agricultural Sciences, Beijing. Animal experiments were performed in accordance with the guidelines established by the Institutional Animal Care and Use Committee at Chinese Academy of Agriculture sciences.

### Isolation and culture of DMSCs

The skin was isolated from the dorsal of the bovine fetus and rinsed 6–10 times in PBS, and digested for 12 h at 4°C using 0.25% collagenase type II. After rinsing the digested skin tissues 6–10 times in PBS, the epidermis tissues were gently scraped off, and rinsed 3–5 times in PBS with 1% (w/v) penicillin and streptomycin (Bioss). The remaining derma was cut into about 1 mm^3^ pieces using an ophthalmic scissors, and digested for 15 min at 37°C with 0.25% (w/v) Tyrisin (Gibco) [containing 0.01% (w/v) EDTA]. Then DMEM (Dulbecco's modified Eagle's medium) (Gibco) containing 10% (v/v) FBS (fetal bovine serum, Hyclone) was added to terminate the reaction. The cell suspension was centrifuged at 100 ***g*** for 8 min, the cells were resuspended with complete medium [(DMEM/F12+ 10% FBS +10 ng/ml bFGF (basic fibroblast growth factor, Peprotech)+2 mM/ml L-Gln (Sigma)] glutamine and seeded in a cell culture dish. Cells were cultured in a 5% (v/v) CO_2_ incubator at 37°C for 2 h, and then the cell suspension was transferred to 6-well plates, and continued to culture at 37°C in 5% CO_2_. When the cells reached 80–90% confluence, 0.25% trypsin and 0.02% EDTA were added to the digested cells and subcultured at a ratio of 1:1. The morphology and growth situation of cattle DMSCs was observed by an inverted microscope.

### Growth kinetics

The cells of P3, P12 and P21 were plated to a 24-well plate with a density of 1.0×10^4^/ml. Viable count were detected by Trypan Blue (Sigma) exclusion test and counting were performed on three wells every day and continually for 8 days. Cell counting per well was repeated for three times to calculate the mean. The PDT (population doubling time) was calculated based on the formula PDT=(t−t_0_) lg2/(lgN_t_−lg), where t_0_ is the starting time of the culture; t the termination time of the culture; N_0_ the initial cell number of the culture; and N_t_ the ultimate cell number of the culture.

### Immunofluorescence staining

The DMSCs of passages 3 were subcultured on a 24-well plate, the cells were fixed in 4% (w/v) PFA (paraformaldehyde) for 15 min and then washed with ice-cold PBS three times (5 min each). Cells were permeabilized by 0.25% (v/v) Triton X-100 (Sigma) for 10 min. The cells were then washed three times (5 min per wash) with PBS and incubated with goat serum (Zhongshan Golden Bridge) at room temperature for 30 min. Then we added anti-CD29 (1:100, sc-53711, Santa Cruz) and anti-CD44 (1:100, ab19622, Abcam), and incubated the cells overnight at 4°C. The primary antibody was removed and cells were washed three times (5 min per wash) with PBS. We then added FITC-conjugated goat anti-mouse or FITC-conjugated goat anti-rat antibodies (Zhongshan Golden Bridge) and incubated the cells at room temperature in the dark for 1 h. The plate was washed three times (5 min per wash) with PBS in the dark. Finally, the cells were incubated with 10 μg/ml DAPI (4′,6-diamidino-2-phenylindole) for 15 min and then washed three times with PBS. Images were obtained using a laser-scanning confocal microscope. Ten randomly selected non-overlapping fields of vision were observed and photographed (Nikon).

### RT–PCR (reverse transcription–PCR) assays

Total RNA of DMSCs at P3, P12 and P21 passages was extracted using Trizol reagent (Invitrogen). Total RNA was reversed transcribed into cDNA, followed by 30 PCR cycles using an RNA PCR kit version 3.0 (Takara). RT–PCR primers of DMSC's surface markers are listed in Table 1. PCRs were operated in 25 μl volumes containing 2.5 μl of 10× PCR buffer (Takara), 16.75 μl of double-distilled H_2_O, 0.25 μl of Ex-Taq (Takara), 1.0 μl each of forward and reverse primers and 1.5 μl of template cDNA. Cycling conditions contained an initial denaturation step at 94°C for 5 min, then 30 cycles at 94°Cfor 30 s, 50–60°C for 30 s and 72°C for 30 s. PCR products were visualized by electrophoresis on 2% (w/v) agarose gels.

**Table 1:** RT-PCR primers sequences of DMSCs

Gene	Direction	Primer sequences (5′→3′)	T_m_ (°C)	Product length (bp)	Cycle number
CD44	Forward	CGGAACATAGGGTTTGAGA	56	301	30
	Reverse	GGTTGATGTCTTCTGGGTTA			
CD29	Forward	GAAACTTGGTGGCATCGT	58	493	30
	Reverse	CTCAGTGAAGCCCAGAGG			
CD73	Forward	CAATGGCACGATTACCTG	56	428	30
	Reverse	GACCTTCAACTGCTGGATA			
CD34	Forward	CCTCATCAGCTTTGCGACTT	60	314	30
	Reverse	CCAGGAGCAAGGAGCACA			
CD90	Forward	CTACCCAACCTTCTACTCAA	56	221	30
	Reverse	TTCACATCCAGGAGGTTC			
CD106	Forward	GTGAAGGCATTAACCAGG	55	379	30
	Reverse	GCACAATAGAGCACGAGA			
CD166	Forward	TATCAGGATGCTGGAAAC	55	498	30
	Reverse	TAGCCAATAGACGACACC			
OCT-4	Forward	CTACCCAACCTTCTACTCAA	58	343	30
	Reverse	TTCACATCCAGGAGGTTC			
GAPDH	Forward	GGCAAGTTCAACGGCACAGTCA	60	364	30
	Reverse	TAAGTCCCTCCACGATGCCAAAG			
Collage type I	Forward	AGAAGCATGTCTGGGTAGGAG	57	358	30
	Reverse	AGGATAGGCAGGCGAGATR			
Osteopontin	Forward	CCAATGAAAGCCCTGAG	59	310	30
	Reverse	TCCTCCTCTGTGGCATC			
PPAR-γ	Forward	ATCCCTGTTCCGTGCTG	56	356	30
	Reverse	GGGATACAGGCTCCACTT			
LPL	Forward	GAACTGGATGGCGGATG	59	256	30
	Reverse	CTGGATTCCGATACTTCGACCT			
Nestin	Forward	ATCCCTGTTCCGTGCTG	59	204	30
	Reverse	GGGATACAGGCTCCACTT			
MAP2	Forward	GAACTGGATGGCGGATG	60	303	30
	Reverse	CTGGATTCCGATACTTCGACCT			

### *In vitro* differentiation of DMSCs

DMSCs from passages 3, 12 and 21 were induced to differentiate into osteoblasts by culturing in L-DMEM with 10% FBS and 10^−7^ M hexadecadrol, 10 mM 2-glycerophosphate and 50 mg/l ascorbic acid for 2 weeks. For adipogenic differentiation, cells were exposed to L-DMEM supplemented with 10^−6^ M hexadecadrol, 10 mg/l insulin, 0.5 mM IBMX (isobutylmethylxanthine) and 10^−2^ M indomethacin (Sigma) for 7 days, with the induction medium changed every 2 days. For neural differentiation, cells were induced with 20% FBS, 10 μM 2-mercaptoethanol (Sigma) for 24 h, washed three times with PBS and then treated with serum-free medium containing 2% (v/v) DMSO (Sigma) and 1 μM butylated hydroxyanisole (Sigma) for 4 h. After induction, cells were subjected to immunofluorescence techniques and RT–PCR assays. Alizarin Red staining was used for the detection of calcium nodules. Oil-Red-O staining was used for the detection of accumulated oil droplets. Gene-specific primer pairs are listed in Table 1.

## RESULTS

### Morphology observation

**Figure 1:**
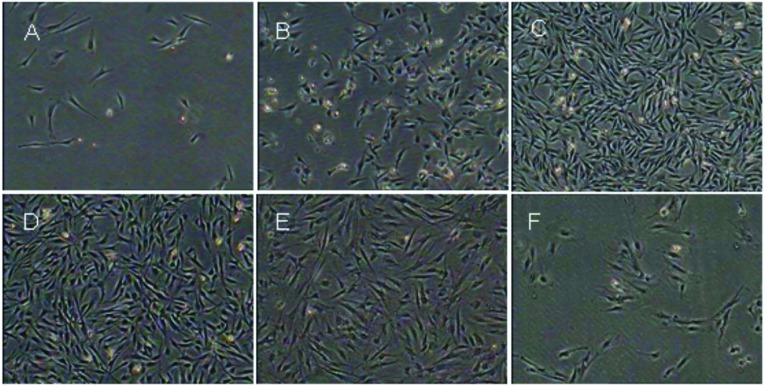
Morphological observations of DMSCs (**A**) Primary cultured cells after 24 h, less cells adhered and stretch. (**B**) DMSCs began to proliferate 48 h later and exhibited a fibroblast-like morphology. (**C**) About 5–6 days later, cells were 80–90% confluent and arranged in a swirl-like pattern. (**D**) The morphology of DMSCs after P4 was uniform, and there were no obvious differences. (**E**) P22 displayed senescence phenomenon. (**F**) P28 shown signs of vacuolization and karyopyknosis.

Primary cultured cells partly adhered to the bottom of the culture plate after 24 h, and began to proliferate 48 h later. Cell morphology was shuttle-like as the cells expanded rapidly. Approximately 5–6 days later, when the density of the swirl-like cells were 80–90% confluent, they were subcultured using 0.25% trypsin (Gibco) and 0.02% EDTA. After three to four passages, the cells’ morphology were uniform, and there were no apparent changes until the 19th passage. Cells were cultured up to the 22nd passage, and most of the cells showed signs of senescence, such as vacuolization and karyopyknosis (Figure 1). As the passage number increased, we observed more cells disappearing from the culture plates.

### Growth kinetics

**Figure 2:**
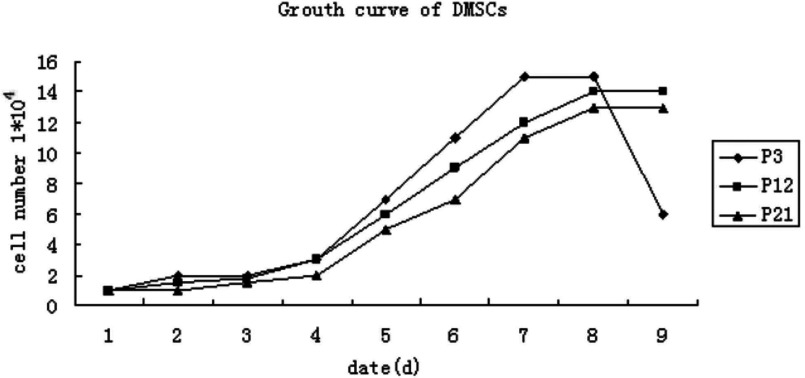
Growth curves of bovine DMSCs from P3, P12 and P21 The growth curves had a typical sigmoidal shape, consisting of latency phase, logarithmic phase and plateau phase.

The cell viability detection was done using hemocytometer and Trypan Blue exclusion test; the growth curves were drawn according to the living cell values. The growth kinetics of BMSCs from P3, P12 and P21 are shown as the growth curves of the figure, which are typically sigmoidal (Figure 2). The whole period of cell growth and proliferation was experienced by the latency phase, the logarithmic phase and the plateau phase. After the latency phase of 72 h cell growth entered the logarithmic phase, and reached plateau phase at about 160 h. The PDT was approximately 72 h. The cattle DMSCs displayed a strong and steady proliferative potential when cultured *in vitro*.

### Bovine BMSCs surface marker detection

**Figure 3:**
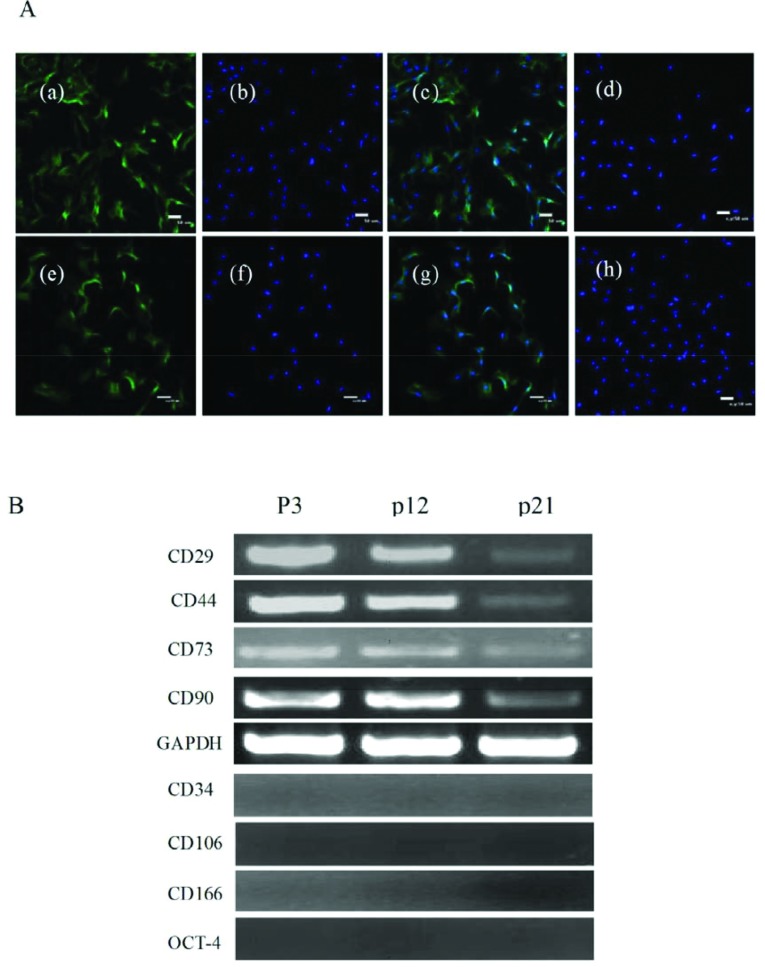
Characteristics of DMSCs surface antigens (**A**) Immunofluorescence analysis (a–c) CD29 positive cells (scale bar=50 lm). (e–g) CD44 positive cells (scale bar=50 lm). (d, h) CD29,CD44 negtive control. (**B**) RT–PCR analysis showed that DMSCs at different passages express CD29, CD44, CD73 and CD90. GAPDH (glyceraldehyde-3-phosphate dehydrogenase) as the internal control, the express of CD34, CD106, CD166 and OCT-4 was negative.

The specific surface antigen markers of bovine DMSCs were detected by immunofluorescence and RT–PCR assays. The results of immunofluorescence staining showed that the BMSCs were CD29 and CD44 positive (Figure 3A). The results of RT–PCR indicated that DMSCs expressed CD29, CD44, CD73 and CD90 (Figure 3B), and the expression of CD34, CD106, CD166 and OCT-4 were negative.

### *In vitro* differentiation of DMSCs

#### Osteogenic differentiation

**Figure 4:**
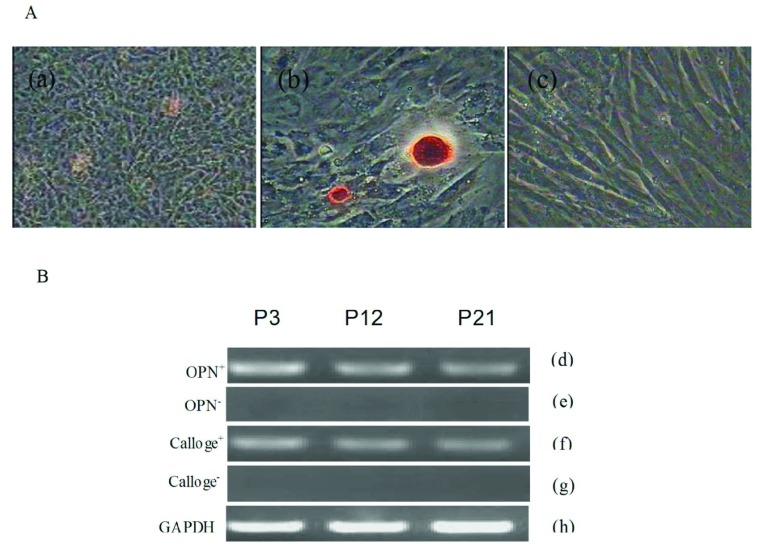
Osteogenic differentiation of DMSCs (**A**) (a) Following induction cell morphology had larger and more apparent nodules as induction time increased. (b) Alizarin Red staining at day 14 post-induction. (c) Control group. (**B**) RT–PCR assays shown expression of osteoblast-marker genes, including collagen type I, osteopontin, at day 14 post-induction. (d, f) Express osteogenic differentiation marker genes collagen type I, osteopontin. (e, g) Negative control displayed the osteogenic differentiation marker genes were negative. (h) GAPDH as internal control

The DMSCs were incubated in osteogenic medium for 14 days, and morphological changes were observed. The morphology change of the cells was from fusiform to tri-dimensional. The cells gathered and formed mineralized nodules with increasing culture time. Furthermore, the nodules were stained by Alizarin Red, the result was positive (Figure 4A). In addition, if the DMSCs were continuously induced, the nodules would increase and grow in size. As control, the morphology of the cells cultured in complete medium did not changed and Alizarin Red staining was negtive (Figure 4A). Osteogenic differentiation of the bovine BMSCs was analysed by RT–PCR (Figure 4B). The osteogenic cell maker genes, including collagen type I and osteopontin, were all expressed in the induced group; there is no evident difference among three different passages.

#### Adipogenic differentiation

**Figure 5:**
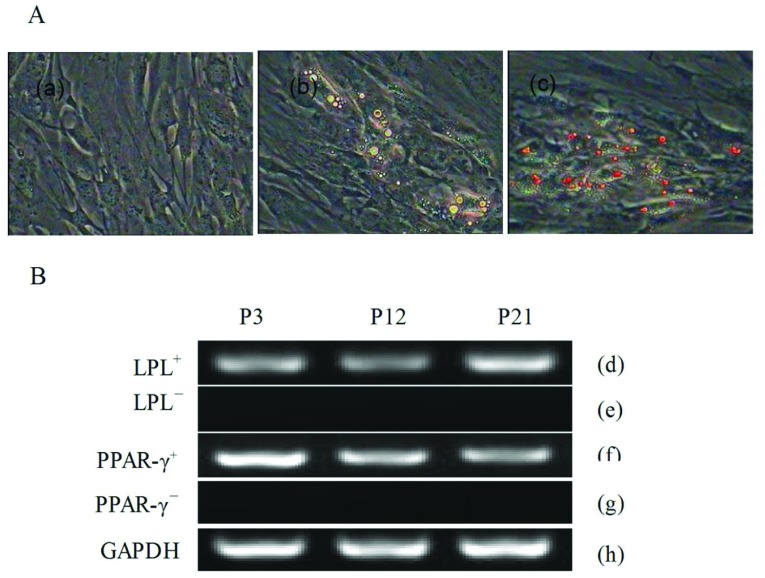
Adipogenic differentiation of DMSCs (**A**) (a) The control group cells staining by Oil-Red-O (200×). (b) The cells after incubation in adipogenic medium for 2 weeks, which had formed lipid droplets (200×). (c) The induced cells staining by Oil-Red-O, the lipid droplets were stained into red (200×). (**B**) RT–PCR analyses of the adipogenic differentiation specific genes, PPAR-γ and lipoprotein lipase. (d), (f) Induced group shown the adipogenic differentiation marker genes were positive. (e, g) Control group shown the adipogenic differentiation marker genes were negative. (h) GAPDH as internal control.

Cells from passages 3, 12 and 21 were plated and grown to about 70% density and then the adipogenic differentiation culture medium was added. Adipogenic differentiation of DMSCs was demonstrated by positive Oil-Red-O staining of intracellular lipid droplets (Figure 5A). The morphology of DMSCs was altered from the fibroblast-like to oblate after 5 days of induction, lipid droplets were gradually observed in the cytoplasm. The number of lipid droplets increased as the induction time increased. Additionally, the expression of osteoblast marker genes, PPAR-γ (peroxisome-proliferator-activated receptor γ) and LPL (lipoprotein lipase) was analysed by RT–PCR (Figure 5B).

#### Neural differentiation

**Figure 6:**
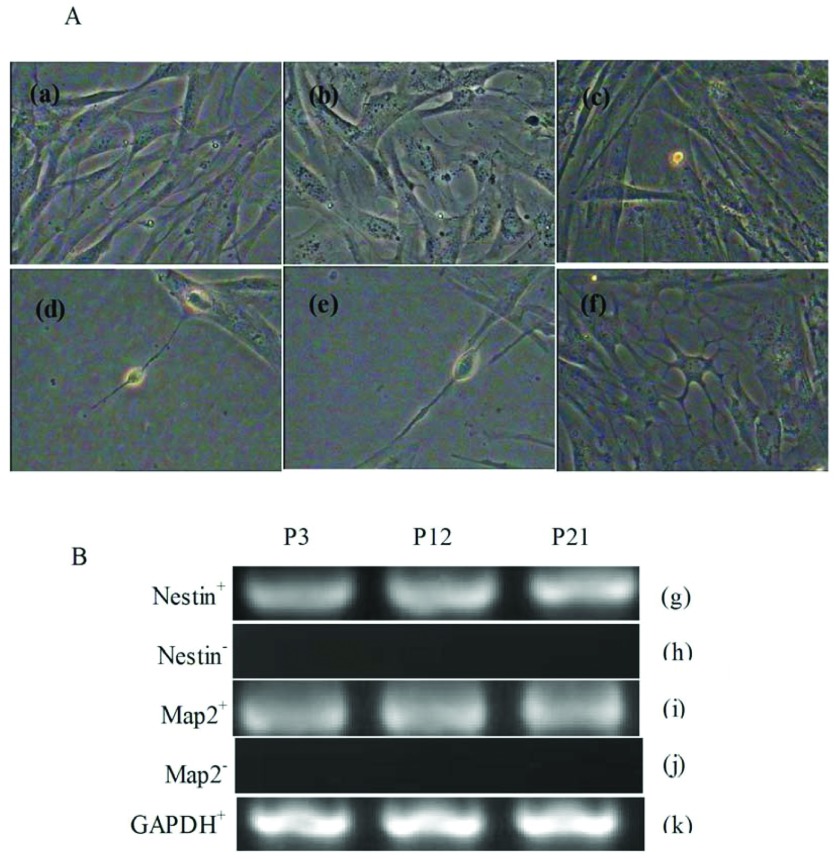
Neural differentiation of DMSCs (**A**) Morphology observe (a–c) Control group, P3, P12 and P21 cell morphology (200×). (d–f) Experimental group, P3, P12, P21 cell morphology (200×), Long axons were observed and cells displayed a typical neuron-like morphology. (**B**) RT–PCR analysis of specific neural markers nestin and MAP-2. (g, i) Induced group shown the neural differentiation marker genes were positive. (h, j) Control group shown the neural differentiation marker genes were negative. (k) GAPDH as internal control.

In the pre-induced DMSCs after 24 h it was observed that spindle-shaped cells began to contract and formed irregular shapes. Following induction, the cell bodies gradually contracted and became round, triangular or cone-shaped with multipolar processes. It was viewed that the process continued to grow with many branches forming and cone-like terminal expansions. A number of cells demonstrated very long processes, which appeared similar to the long axon of neurons (Figure 6A). RT–PCR results showed that the markers of neural cells nestin and MAP-2 (microtubule-associated protein 2) were expressed in the differentiated cells (Figure 6B).

## DISCUSSION

In this study, DMSCs were isolated from 3- to 4-month-old Simmental cattle. The self-renewal and differentiation abilities of DMSCs were also estimated. The age of the fetal bovine played an important role in when the primary cell was obtained; we generally took 3–4 months old fetal bovine as the research object. We used enzymatic digestion [13] to isolate DMSCs from Simmental cattle, the primary cell culture fluid was replaced to remove the non-adherent cells, the main morphology of adherent cells was spindle-like and minority morphology was triangular. We believed that enzymatic digestion was a more advantageous method for obtaining DMSCs from the dermal tissues.

So far, the specific markers of DMSCs were not found, so the general surface markers of mesenchymal stem cells, such as CD29, CD44, CD73 and CD90 were detected to identify the surface antigen. As control, the specific markers of the haematopoietic stem-cell surface antigens such as CD34, CD106, CD166 and OCT-4 were detected to remove the false positive cells. CD29 is an integrin subunit associated with later-stage antigen receptors. Integrin family members are membrane receptors, which are necessary for cell adhesion and recognition in a variety of processes including embryogenesis, haemostasis, tissue repair and metastatic diffusion of tumor cells [14]. The CD44 protein is a cell-surface glycoprotein involved in cell–cell interactions, cell adhesion and migration. It is a receptor for HA (hyaluronic acid) and can also interact with other ligands, such as osteopontin, collagens and MMPs (matrix metalloproteinases). The CD44 protein participates in a wide variety of cellular activities including recirculation and homing, lymphocyte activation and metastasis [15]. CD73 is a plasma membrane protein, it catalyses the conversion of extracellular nucleotides to membrane-permeable nucleosides [16]. The encoded protein is used as a determinant of lymphocyte differentiation. CD90 is Thy-1 cell surface glycoprotein antigen, CD90 (Thy-1) is particularly abundant on the surface of mouse thymocytes and peripheral**** T-cells, and is often used as a marker in adoptive transfer experiments to distinguish between the donor and the recipient T-cells with different CD90 subtypes [17].

Stem cells are a kind of pluripotent cells, it can differentiate into various functional cells under certain conditions. DMSCs are typically characteristic of stem/progenitor cells from different tissues that have the ability to differentiate into cells of any germ layer. So under different induction conditions, DMSCs can differentiate into other kinds of cells. DMSCs originate from the mesodermal [18], but can be differentiated *in vitro* into entoderm and ectodermal cells such as osteocytes, adipogenic and neural cells. However, differentiation mechanisms of DMSCs *in vitro* have not been made clearly. If their differentiation mechanisms is further researched, we will make greater progress in stem-cell therapy.

In recent years, stem-cell therapy has become a potential tool for tissue repair and regeneration [19], particularly dermal matrix with dermal mesenchymal stem cells have been attraction attention. The investigation shows that an artificial dermal matrix has been used as a dermal template for skin regeneration, to form a multifunctional scaffold with hMSCs (human bone marrow-derived mesenchymal stem cells) and PRP (platelet-rich plasma) for tissue engineering and regenerative technology [20]. Autograft is the gold standard for full thickness burns, but there is a lack of suitable donor sites and poor skin quality, so skin substitutes has been attracting great attention [21]. Combining mesenchymal stem cells with artificial dermal substitutes may show an important potential for treating full thickness burns, even in allogeneic combinations due to the immunoregulatory property of these cells [22].

### Conclusion

In this study, we successfully isolated and cultured DMSCs from the Simmental cattle dermal tissue, and identified cells in cell morphology and molecular biology. The self-renewal ability and differential potential of DMSCs were assessed *in vitro*. We demonstrated that DMSCs can be induced to osteogenic, adipogenic and neural cells, which proved that DMSCs are multipotential stem cells. Through this study, we have not only provided a fundamental technology platform for the establishment of cattle dermal mesenchymal stem cells bank, but also provided an important and a new way for the potential application of DMSCs as a stem-cell source for regenerative therapies.
